# Integrative systematic review of psychodrama psychotherapy research: Trends and methodological implications

**DOI:** 10.1371/journal.pone.0212575

**Published:** 2019-02-19

**Authors:** Hod Orkibi, Rinat Feniger-Schaal

**Affiliations:** School of Creative Arts Therapies, Faculty of Social Welfare and Health Sciences, University of Haifa, Haifa, Israel; University of California Los Angeles, UNITED STATES

## Abstract

**Background:**

Psychodrama is an experiential psychotherapy in which guided role-play is used to gain insights and work on personal and interpersonal problems and possible solutions. Despite the wealth of literature describing clinical work, psychodrama intervention research is relatively scarce compared to other psychotherapies and psychological interventions.

**Objective:**

For this reason we implemented the integrative approach to systematic review that authorizes the combination of publications with diverse methodologies and all types of participants, interventions, comparisons, and outcomes. Our aim was to produce a comprehensive summary of psychodrama intervention research in the last decade that critically evaluates methodological issues to inform future studies.

**Methods:**

We searched four major electronic databases (PsycINFO, PubMEd, Scopus by Elsevier, and Web of Science) for peer-reviewed articles on psychodrama interventions published in English between 1 January 2007 and 31 December 2017. The quality of qualitative and mixed methods studies was assessed on the basis of pre-established guidelines, and the risk of bias was assessed for all quantitative randomized control studies, consistent with the PRISMA protocol.

**Findings:**

The database search and a hand search resulted in 31 psychodrama intervention publications. Overall, these studies examined the effects of psychodrama on more than 20 different outcomes and most studies had adult clients. The next largest group was adolescents, whereas only two studies involved children. Thus psychodrama intervention research in the last decade suggests there are promising results in all methodologies, and highlights the need to enhance methodological as well as reporting quality and to theorize and examine modality-specific mechanisms that lead to therapeutic change. Recommendations to improve methodology, transparency, and specificity in reporting future psychodrama and other psychotherapy research are discussed.

## Introduction

Psychodrama (PD), originated in 1921 by J. L. Moreno, is an experiential psychotherapy in which clients use guided role-play to work on their personal and interpersonal problems and possible solutions through actions rather than words alone [1, 2]. PD offers clients a “fail-safe” reality where feelings, thoughts, and behaviors can be explored and insights can be gained into past issues, present challenges, and future possibilities. The specificity of PD is that clients are encouraged to express their feelings directly and in the first person, to talk *to* rather than *about* their significant others (who are normally played by a group member or represented by an empty chair), which facilitates high levels of in-session experiencing. Although Moreno developed PD based on his group work and interactions with children using storytelling and role-play [1], today PD is used with clients of all ages, with individuals, couples, and families [3].

Despite the wealth of literature describing clinical work with clients of all ages and different problems, PD intervention research is relatively scarce compared to research on other psychotherapies. The focus in the PD literature has mostly been on describing and explaining processes through anecdotal experiences, clinical vignettes, and case illustration reports. Some of the key obstacles to conducting research are the predominantly clinical orientation of most PD practitioners and the related fact that almost all training programs take place in private rather than research institutions and are thus more experiential and clinical than research-oriented.

In the PD literature, three major systematic reviews of research have been published. In Kellermann’s review of 23 outcome studies (articles published between 1952 and 1985) he concluded that PD “is a valid alternative to other therapeutic approaches, primarily in promoting behavior change with adjustment, antisocial, and related disorders” [4]. Kipper and Ritchie [5] conducted the first meta-analytic study that focused on the effectiveness of using specific PD techniques in 25 experimentally designed studies (articles published between 1965 and 1999). The analysis revealed “an overall effect size that points to a large size improvement effect similar to or better than that commonly reported for group psychotherapy in general [of 0.50, and] the techniques of role reversal and doubling emerged as the most effective interventions” [5]. Wieser published the latest descriptive review of 52 studies (articles published between 1948 and 2006) on the treatment effects of PD, and concluded that “there is still a need for basic research into the effectiveness of psychodrama therapy” [6].

### Types of review methods

There are several kinds of research review methods. The most basic is the simple *literature review* that only summarizes the literature on a given topic in narrative form and does not necessarily follow rigorous and explicit strategies for search, selection, or analysis of the literature [7]. A more in-depth research review method is the *systematic review* that implements an explicit method for conducting a comprehensive search, and for selecting and evaluating previous quantitative studies on a well-defined clinical question (e.g., does PD reduce youth depression?). Systematic reviews include narrative analysis of the primary studies and, sometimes, descriptive statistics and quantification of qualitative information. They provide reliable evidence on gaps in current research, solid grounds for determining evidence-based practice, and the first stage of meta-analysis studies [8]. While systematic reviews have traditionally focused predominantly on questions relating to the effectiveness of interventions, there are at least 10 different types of systematic reviews that address diverse questions and the “information needs of healthcare professionals and policy makers” [9]. One type, which is particularly pertinent to this study, is a methodological systematic review that examines methodological issues relating, for example, to design and conduct. A *meta-analysis* is considered the highest level of evidence and the gold standard of literature-based research, where carefully selected data from primary quantitative studies are combined with rigorous statistical procedures to calculate an overall effect size [10].

Nevertheless, given the relatively small number of PD studies, we implemented an *integrative review* approach that allows for the combination of publications with diverse methodologies including quantitative, qualitative, and mixed methods [11]. This review approach can produce a more comprehensive portrayal of research trends in a given field. As such, integrative reviews can help generate new knowledge, as well as inform research, practice, and policy initiatives [12].

### The present study

Drawing on a recent typology of systematic reviews in the medical and health sciences, the present study is an integrative methodological systematic review [9]. Hence the overarching question in this review was to determine which trends and methodological issues could be identified in psychodrama psychotherapy research. Accordingly, this systematic review had two aims: (a) to summarize 10 years of PD intervention research with all types of participants, interventions, comparisons, and outcomes according to the eligibility criteria described below; and (b) to critically evaluate methodological issues to help determine the requirements for future studies.

The present study is part of a wider project reviewing both PD and drama therapy research [13]. But because these two treatment modalities are considered different healthcare professions, two distinct reports were generated. Briefly, in psychodrama, clients typically use role-play to enact themselves, parts of themselves, or significant others in their real lives, and hence work more directly on reality-based issues. In contrast, drama therapy is more fantasy-based and clients typically use role-play to enact fictional and symbolic roles, use storytelling, puppetry, masks, and miniature objects to work more indirectly and with greater dramatic distance from their issues. However, some contemporary variants of psychodrama practice involve working with metaphors and imagination, thus blurring the boundaries between classical PD and drama therapy [14–16].

## Methods

We systematically searched for studies using drama as a therapeutic modality, both PD and drama therapy, in four major electronic databases: PsycINFO, PubMEd, Scopus by Elsevier, and Web of Science. Search areas were title, abstract, and keywords. The search terms for all the databases were: *Psychodrama* OR *dramatherapy* OR *drama therapy* AND *intervention* OR *program* OR effect* OR evaluat* OR “*case study*.” In addition, we conducted a hand search in the following three journals because they are specific to the treatment modalities: *Drama Therapy Review*, *Dramatherapy* (published by the British Association of Dramatherapists), and *The Journal of Psychodrama Sociometry and Group Psychotherapy* (published by the American Society of Group Psychotherapy and Psychodrama). We also searched a special issue on Psychodrama published in the *International Journal of Psychotherapy*. Note that the Australian and Aotearoa New Zealand Psychodrama Association Journal was excluded because the journal’s Guidelines for Contributors do not indicate a formal peer review process. In addition, the Zeitschrift für Psychodrama und Soziometrie (Journal for Psychodrama and Sociometry) was excluded because most of its papers are in German. The British Journal of Psychodrama and Sociodrama was also excluded because it is only available in print and is not indexed.

### Data extraction

A spreadsheet for data extraction was piloted and then used to record information of interest and the PICOS framework specifying participants, interventions, comparisons, outcomes, and study designs [17]. Information was extracted from each study on: (1) study method: quantitative, qualitative, mixed methods; (2) study design (e.g., randomized / nonrandomized group comparison pre-post, single group pre-post, single subject design, and follow up); (3) characteristics of participants: *N*, age, gender, diagnosis, setting, country; (4) intervention type (duration, frequency, structure, protocol) versus control or placebo; (5) primary outcome measures; (6) results (significance, effect size). The authors of five studies were contacted with questions to verify information about their study [18–23].

### Eligibility criteria

Eligibility criteria were for the publication to be an intervention research with quantitative and/or qualitative data, written in English, and published in a peer reviewed journal. No restrictions were imposed on publication status. Publication date was restricted to between January 2007 and December 2017 (the date last searched was 10 January 2018). Eligibility assessment was performed independently by the two authors and cases of disagreement were discussed until all issues were resolved and there was consensus.

### Assessment of qualitative and mixed methods studies

The assessment of the qualitative and mixed methods studies drew on Hawker and colleagues’ tool for critically and systematically reviewing research conducted using different paradigms [24]. Consistent with the purpose of this review, we focused on the seven methodological dimensions defined by Hawker et al.: method and data, sampling, data analysis, ethics and bias, findings, and transferability. Three dimensions that do not address methodological issues are not reported in this review (i.e., “abstract and title,” “introduction and aims,” “implications and usefulness”).

### Risk of bias assessment

Consistent with the Preferred Reporting Items for Systematic Reviews and Meta-Analyses (PRISMA) [25], we used the Risk of Bias (RoB) assessment tool that was developed by the Cochrane Collaboration for randomized controlled trials [26]. Generally, RoB assessment focuses on the internal validity of a study, and in particular on methodological flaws that can lead to bias. Because the results of a study may in fact be unbiased despite a methodological flaw, the tool assesses the *risk* of bias. Bias is assessed in several domains for which there is empirical evidence that biasing can influence the estimates of an intervention’s effectiveness. A bias can lead to underestimation or overestimation of the true intervention effect [25].

Recently, it has been noted that because the RoB tool was developed within the field of medicine, its application to the context of psychotherapy outcome research requires several adjustments [27]. Drawing on the adjusted criteria suggested in Munder and Barth, we assessed the following six potential sources of bias: (1) random sequence generation, (2) concealment of allocation to conditions, (3) blinding of participants and personnel to condition assignment, (4) handling of incomplete outcome data, (5) selective outcome reporting, and (6) treatment implementation. For each source of bias, two raters judged whether the risk of bias was *low*, *unclear*, or *high* according to definitions and examples in Munder and Barth (2018). Note that because all the primary outcomes in the studies were self-reported and because in psychotherapy it is impossible to blind patients to intervention aims and content we did not code RoB given the absence of blinding of the outcome assessors [28]. Hence, the RoB tool was only used for the assessment of studies with a randomized group comparison design.

## Results

**Fig 1:**
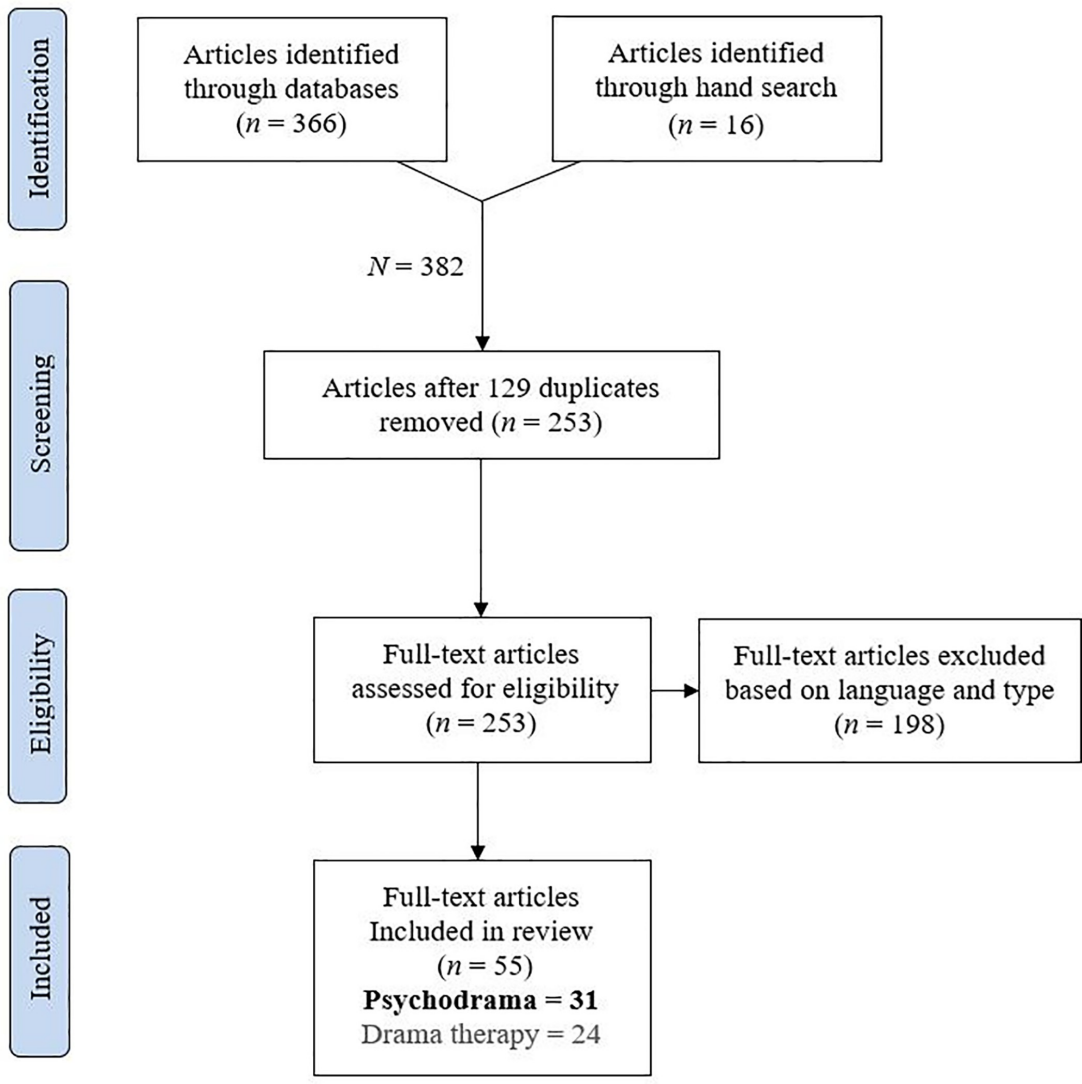
Systematic search according to the PRISMA statement methodology.

This article follows the reporting guidelines of the PRISMA framework where possible [25]. Fig 1 presents a PRISMA flow diagram indicating the studies retrieved for the review, screening, and assessment of eligibility (S1 Table).

We identified 162 articles through Scopus and 113 through PsycINFO. The Web of Science and PubMEd searches yielded 66 and 25 articles, respectively. Additionally, 16 articles were found through hand search and a reference list search (total initial *N* = 382). Within these results, we searched for duplications and excluded 129 articles, yielding 253 articles after duplicates had been removed. Next, 201 articles were excluded based on full-text examination of publication type and the language of main text. Specifically, we excluded articles that were review articles, book reviews, media reviews, editorials, obituaries, and case studies/ examples/ illustrations/ vignettes. Because we focused on intervention research, articles that only described techniques or treatment programs without testing their effect on participants were also excluded. Similarly, articles concerning therapists, caregivers, teachers, or students in training rather than therapy clients were also excluded. We excluded articles where the word “drama” was used vaguely without specific indication of PD or drama therapy, and articles with eclectic treatments where PD or drama therapy were not indicated as the primary approaches. We also excluded several articles that were automatically detected because their Abstract was in English but the main text was in a different language (Persian, Croatian, Turkish, Polish, Portuguese, German, and French). One study was excluded because it could not be obtained. In total, 55 articles met the eligibility criteria, of which 31 were PD articles (60%) and 24 were drama therapy articles. This review focuses on the 31 PD articles presented in Tables 1 to 4 (the drama therapy articles are reviewed in–masked for the review, 2018).

**Table 1:** Characteristics of the qualitative studies.

First Author (year) country	*N*	Clients	Setting	Intervention (protocol)	Data collection and analysis	Primary Findings
Alby et al. (2017) Italy	10	Adults w. cancer & family members (*M*_age_ = 57)	University (recruited from a hospital)	Group analytic PD, 1.5h semimonthly, 2 years (analytic three phases)	Interviews & thematic analysis	Increased self-awareness, expresses and shares feelings, reduces fears, aids decision making
Konopik (2013) USA	13	Adults w. MHC (*M*_age_ = 40)	Hospital	Group PD, 2h weekly, 4–6 sessions (structured five stages)	Observational, interviews & communication content analysis	Changes in emotions, family of origin issues, self-awareness and self-worth, shift in views
Menichetti (2016) Italy	8	Adults w. cancer (*M*_age_ = 59)	Hospital	Group PD, 1.5h weekly, 1–80 sessions (three PD phases)	Interviews & Interpretative phenomenological analysis	Self-expression, ability to give and receive help, sense of agency, cope with grieving
Vural (2014) Turkey	7	Mothers of children with ADHD (*M*_age_ = NR)	Special education center	Group PD, 2h weekly, 12 sessions (semi structured)	Observational & analysis of participants in-session statements	Instilled hope, self-confidence, better coping, reduced anger and punishment

PD = psychodrama. MHC = mental health conditions. ADHD = Attention deficit hyperactivity disorder. NR = not reported.

**Table 2:** Characteristics of mixed methods studies.

First author (year) country	*N*	Clients	Setting	Intervention (protocol)	Design	Primary outcomes	Qualitative findings
Chae (2017) South Korea	17	College students w. difficulties (M_age_ = NR)	University	1 Group PD, 2h, 10 sessions in 2 weeks (three PD phases)	Single group, pre-post Qual: post treatment feedback and interviews	Reduced insecure attachment: avoidance & anxiety	More positive view of self, others, and relationships
Dogan (2010) Turkey	21	Masters’ students w. interpersonal difficulties (M_age_ = 25)	University	2 Groups: PD vs. placebo on effective learning, 2h weekly, 12 weeks (three PD phases)	Randomized, pre-post Qual: observation during sessions and last session feedback	No group differences in insecure attachment: avoidance & anxiety	More self-understanding and confidence, insight, hope, relationships
Gatta (2010) Italy	6	Adolescents w. psychiatric disorders (M_age_ = 17)	Semi-residential public service	2 Groups: PD vs. matched coping skills TAU, 1.25h weekly, 12 sessions (semi-structured)	Nonrandomized group comparison, pre-post Qual: case studies	Group difference in some symptoms	Participants’ feedback on the treatment
Harkins (2011) UK	76	Adult inmates (M_age_ = 35)	Prisons	1 Group: PD + CBT, 2–3 days (protocol)	Single group, pre-post & observations Qual: post treatment interviews	Improved self-efficacy, motivation to change, confidence in skills	More confidence and better prepared to cope with the future after release
Karabilgin (2012) Turkey	7	Adults w. HIV/AIDS (M_age_ = 33)	University center	1 Group PD, 10 sessions of 6h (three PD phases)	Single group, pre-post Qual: pre and post treatment focus groups with descriptive analysis	Improved mental health only, out of eight quality of life dimensions	More acceptance and confidence to talk about HIV/AIDS feelings, cope with fear. Less depression
McVea (2011) Australia	17	Adults w. unresolved emotions (aged 27–66)	Private practice	1 Group, 2.5 days workshop (three PD phases)	Single group, pre-post, follow-up Qual: post workshop feedback, BSR, CCI	Gain in in-session resolution, reduction in interpersonal distress, but not in symptoms	Improvements in interpersonal functioning and sense of self
Terzioğlu (2017) Turkey	30	Women w. infertility (M_age_ = 32)	University hospital	2 Groups: PD vs. control NR, 3h weekly, 8 sessions (three PD phases)	Nonrandomized group comparison, pre-post Qual: observation, interview–unclear	Improved self-esteem, less depression, hopelessness. Result reported for anxiety are unclear	Increased awareness, competence, self-worth

PD = psychodrama. Qual = qualitative data collection and analysis. NR = not reported. TAU = treatment as usual. BSR = brief structured recall method. CCI = Client change interview protocol.

**Table 3:** Characteristics of quantitative single group studies.

First author (year) Country	*N*	Clients	Setting	Intervention (protocol)	Design	Primary Outcomes
Akinsola (2013) Nigeria	25	Children with social anxiety (aged 7–16)*	School	Group PD, three days (three PD phases)	Single group pre-post	Decreased social and performance anxieties
Bilge (2017) Turkey	28	University students with anger problems (*M*_age_ = 21)	University	Group psychoeducation & PD, 2h bimonthly, 3 sessions (3-stage protocol)	Single group pre-post	Decreased anger
Biolcati (2017) Italy	30	University students with MHC (*M*_age_ = 22)	University center	Group analytical PD, 1.5h weekly 40 sessions (unclear)	Single group pre-post	Improved well-being, decreased symptoms and risk
Högberg (2008) Sweden	14	Suicidal children and adolescents (*M*_age_ = 15)	Outpatient clinic	Individual DP** 1.5-2h bimonthly *M* sessions *=* 17 (semi-structured)	Single group pre-post + follow-up	Improved global functioning pretest-posttest & pretest-follow-up
Orkibi (2017)^1^ Israel	16	Adolescents at-risk (*M*_age_ = 15)	School	Group PD, 1.5h weekly 16–20 sessions (three PD phases)	Single case design change process	Increased in-session dramatic engagement predicted productive behaviors
Orkibi (2014) Israel	12	Adults with MHC and students (aged 22–60)*	University center	Group PD & theater 2h weekly, 20 sessions (three PD phases)	Single case design	Pretest-posttest & pretest-follow-up decreased public stigma and self-stigma, increase in self-esteem

*Mean age not reported.

** Some sessions included parents. PD = psychodrama. MHC = mental health conditions. NR = not reported. 1 = Orkibi, Azoulay, Regev, et al. (2017).

**Table 4:** Characteristics of quantitative non-randomized and randomized group comparison studies.

First author (year) Country	*N*	Clients	Setting	Intervention (protocol)	Design	Primary Outcomes
**Non-Randomized Group Comparison Studies**						
Oguzhanoglu (2014) Turkey	28	Women with major depression (*M*_age_ = 35)	University polyclinic	3 Groups: PD & med, vs. med only vs. control, 3h weekly, 16 sessions (loose structure)	NRGC pre-post	Similar decrease in depression in PD & med group and in med only group
Orkibi (2017)^1^ Israel	40	Adolescents at-risk (*M*_age_ = 15)	School	2 Groups: PD vs. waiting list control, 1.5h weekly, 16–20 sessions (three PD phases)	NRGC pre-post & process-outcome analysis	Only in PD increases in 3 out of 4 self‐concepts, decrease in loneliness. Some process-outcome correlations
**Randomized Group Comparison Studies**						
Aytemur (2012) Turkey	113	Adult smokers (*M*_age_ = 47)	University clinic	2 Groups: PD & ST vs. ST only 2h weekly, 8 sessions (semi structured)	RGC 3-wave measures	PD increased the success rate of smoking cessation in the early period
Dehnavi (2016) Iran	30	Men with opiate dependence (*M*_age_ = 31–29)	Addiction treatment clinic	2 Groups: PD vs. control 2h, 12 sessions in 6 weeks (three PD phases)	RGC pre-post	Increase in general quality of life
Kähönen (2012) Finland	77	Adults working in public service with burnout (*M*_age_ 47.5)	Private occupational healthcare services	3 Groups: PD vs. analytic vs. control, 6h, 17 days (unclear)	RGP * pre-post + 6-month follow-up	PD group showed a higher increase in sense of coherence than the analytic group during the intervention
Karataş (2011) Turkey	36	High school students manifesting aggression	School	3 Groups: PD vs. placebo of interaction vs. control, 1.5-2h weekly, 10 sessions (three PD phases)	RGC pre-post + 12-week follow-up	PD group higher increase in problem solving and decrease in aggressions, but follow-up only for aggression
Karataş (2014) Turkey	45	University students low on SWB and high on hopelessness (*M*_age_ = NR)	University	3 Groups: PD vs. control vs. placebo of reading, 1.5-2h weekly, 12 sessions (three PD phases)	RGC pre-post + 10-week follow-up	PD group increased SWB and decreased hopelessness compared to control and placebo. Follow-up effect lasted for hopelessness but not SWB
Karataş (2009a) Turkey	23	High school students manifesting aggression (9th grade)	School	2 Groups: PD vs. control 1.5-2h weekly, 14 sessions (three PD phases)	RGC pre-post + 16-week follow-up	PD decreased total aggression, anger, hostility, but not physical and verbal aggression
Karataş (2009b) Turkey	36	High school students with aggression (9th grade)	School	3 Groups: PD vs. CBT vs. control, 1.5-2h weekly, 10–14 sessions (three PD phases)	RGC pre-post + 16-week follow-up	PD and CBT decreased aggression compared to control, but CBT more than PD
Özbaş (2016) Turkey	82	Oncology nurses with burnout (aged 28–37)	Hospital inpatient oncological clinics	2 Groups: PD vs. control 2h weekly,10 sessions (semi structured)	RGC pre-post + 3-month follow-up	PD group decrease burnout and increase psychological and workplace empowerment
Smokowski (2009a,b)^2^ USA	81	Latino immigrant adolescents** (M_age_ = 14)	Community	2 Groups: PD vs. support group, 3h weekly, 8 sessions (protocol)	RGP pre-post + 1-year follow-up	(2009a) Pre-post: group differences were not significant (2009b) Pre-follow-up: PD group maintained superior effects than support group
Sproesser (2010) Brazil	16	Adults with Parkinson's disease (*M*_age_ = 58–60)	Hospital outpatient clinic	2 Groups: PD vs. waiting list control 1.5h bimonthly, 12 sessions (unclear)	RGC pre-post	PD group had stronger improvement in depression, anxiety, and quality of life
Tarashoeva (2017) Bulgaria	40	Adults with panic disorders (*M*_age_ 42)	Private mental health center	2 Groups: PD & med vs. & med only control 3h weekly, 25 sessions (unclear)	RGC pre-post + 6-month follow-up	PD group greater reduction in anxiety symptoms, improved spontaneity, quality of life and social functioning

PD = psychodrama. Med = medication. NRGP = Non-Randomized Group Comparison. RGP = Randomized Group Comparison. ST = standard treatment. SWB = subjective well-being. NR = not reported.

* Randomization was only for treatment groups.

**Some sessions included parents.

1 = Orkibi, Azoulay, Snir, et al. (2017). 2 = the results of the same study were reported in two separate articles.

### General characteristics of psychodrama studies

**Fig 2:**
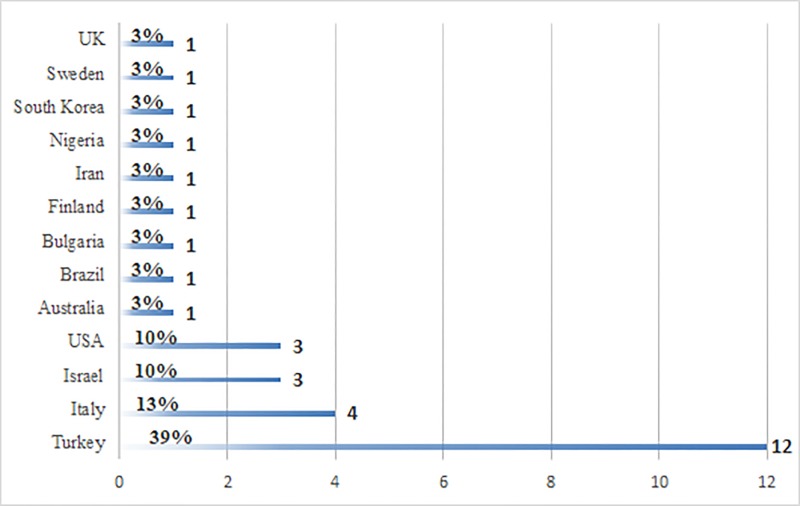
Frequency of included publications by country.

As can be seen in Fig 2, the majority of the PD research publications were from Turkey (*n* = 12, 39%), with the next largest groups from Italy (*n* = 4, 13%) and Israel (*n* = 3, 10%), and USA (*n* = 3, 10%).

The frequency of PD publications per year ranged from one (in 2008) to eight (26% in 2017), with a total average of three publications per year. Of the 31 PD publications, 20 were quantitative, four were qualitative, and seven had a mixed methods design.

**Fig 3:**
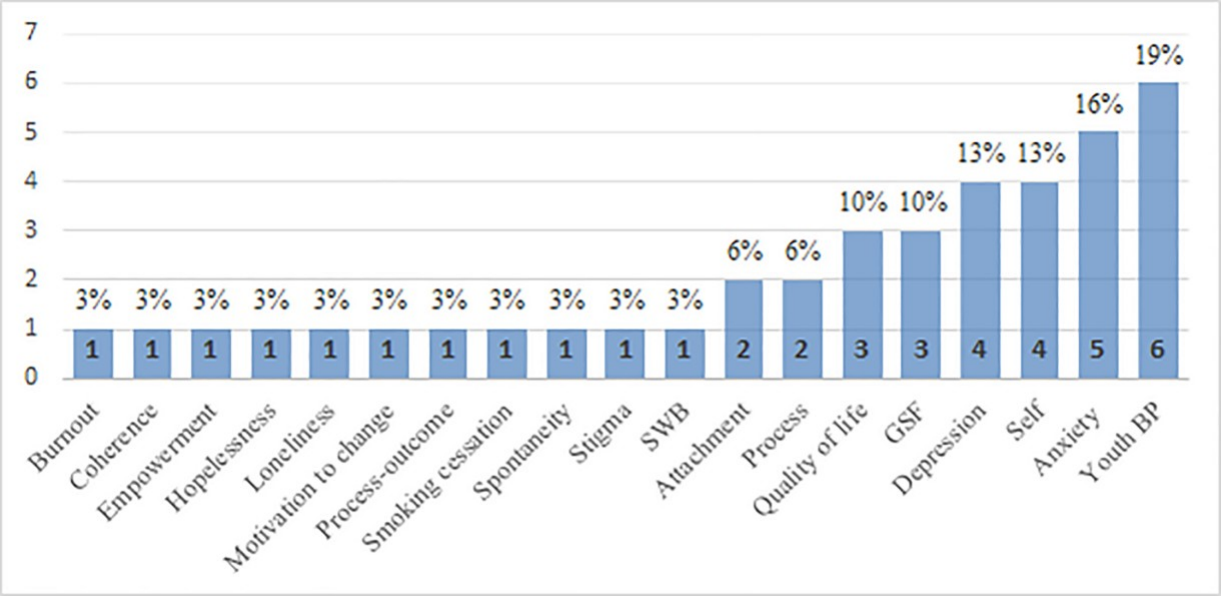
Primary outcomes. Frequency of primary outcomes. SWB = subjective wellbeing, GSF = general symptoms and functioning, BP = behavioral problems.

Regarding client populations and primary outcomes, as can be seen in Fig 3, the majority of the PD research publications dealt with youth at risk (*n* = 10, 32%), followed by students (*n* = 4, 13%), and adults with different mental health conditions (*n* = 4, 13%).

The most frequent outcomes, in descending order, were adolescents behavioral problems (*n* = 6, 19%), followed by anxiety (*n* = 5, 16%) and depression (*n* = 4, 13%). Next was quality of life (*n* = 3, 10%) followed by symptoms and global functioning (*n* = 3, 10%). Two studies measured attachment (6%) and two studies measured in-session process variables (6%), of which one measured dramatic engagement [29]. Only one study measured spontaneity, which is a core construct in PD theory [30]. As also seen in Fig 3, other primary outcomes included subjective well-being, smoking cessation, burnout, hopelessness, loneliness, sense of coherence, public stigma, self-stigma, self-esteem, self-efficacy, and self-concept. The next sections present narrative reviews of the characteristics of these studies as a function of methodology.

### Qualitative studies

All four qualitative studies had a single group design. As seen in Table 1, the number of participants in the qualitative studies ranged from 7 to 13 (*M* = 10, *SD* = 2.6). All the studies involved adults: two studies examined Italian adults with cancer [20, 31], one study dealt with adults with mental health conditions in the USA [32], and one study was about Turkish mothers of children with Attention Deficit Hyperactivity Disorder (ADHD) [33]. Two of the studies dealt with hospital settings [31, 32], one study was conducted in a special education center [33], and one at a university [20].

PD treatment was delivered in a group format in all four studies: one study delivered analytic PD group therapy [20] and the three others followed the “classical” PD approach. In three studies, session duration ranged from 1.5 to 3 hours per week, and the number of sessions ranged from 1 to 80. The exception is the study with the analytic PD group that delivered two 1.5 hours sessions per month, for two years [20]. In terms of session structure, two studies had a structured [32] or a semi-structured format [33], one followed the standard three PD phases of warm-up, action, and sharing [31], and one followed an analytic 3-phase structure that included free associations as the group warm-up phase [20]. Information concerning the qualifications of the therapists was only provided in two studies: “a drama therapist educated in a national psychodrama institution and an assistant therapist” [33] and a “certified psychodrama therapist” [32]. Only one study indicated that the PD therapist received clinical supervision during treatment implementation [33].

Regarding data collection, two studies conducted post-treatment interviews [20, 31], and one study included both post-treatment interviews and direct observation during the sessions [32]. In one study, data were only collected by notes taken during the sessions [33]. Data analysis involved content/ thematic analysis [20, 32], interpretative phenomenological analysis [31], and analysis and quantification of participants’ in-session statements [33]. Detailed and clear descriptions of the data analysis procedures were presented in two studies [31, 32], whereas in one study the description was unclear [33] and in another study minimal details were provided about the data analysis [20].

In most studies, some context and setting were described. The recruitment procedure was reported in all studies, but information on sample demographics was relatively sparse across studies, with one study lacking even the a minimal necessary information such as participating mothers’ age and marital status [33]. Informed consent from participants was only reported in two studies [31, 33] and the issue of protecting participants’ confidentiality and anonymity was mentioned in one study alone [32]. Only one study mentioned the potential influence of the therapists or researchers on the findings, “since the patients who were interviewed were selected in consultation with group facilitators” [31]. There were no other indications of the researchers’ reflexivity regarding their potential influence on the findings in terms of their own bias, perspective, role, and interactions with participants.

Finally, in all studies sufficient data were presented to support the findings, ranging from relatively long quotations [31] to short quotations [20, 32], and paraphrases of the participants’ statements [33]. A procedure to enhance the credibility of the findings was only reported in one study [32], where the themes were “confirmed in consultation with a psychodrama expert.” It is not clear, however, if this expert was blind to the study aims.

### Mixed methods studies

As shown in Table 2, of the seven mixed methods studies, four had a single group design [23, 34–36]. Two studies had nonrandomized group comparison designs: one with a treatment-as-usual control group matched for age, gender and diagnosis to minimize selection bias [37], and one with an unmatched and unspecified control group [38]. One mixed methods study had a randomized group comparison design and a 4-week control group on “effective learning and efficient study methods” which was shorter than the 12-week PD group [39].

The number of participants in the mixed methods studies ranged from six to 76 (*M* = 25, *SD* = 24). Most of the studies involved adult participants. One study involved adult inmates in the UK who were approaching their release date [36], one study involved adults in Turkey with HIV or AIDS [23], one study involved adults in Australia with unresolved painful emotional experiences [34], and one study involved women in Turkey with a diagnosis of infertility [38]. One study involved college students in South Korea struggling with life adjustment and interpersonal relationships [35], one involved Master’s students in Turkey with interpersonal difficulties [39], and one study involved adolescents in Italy with psychiatric disorders [37]. The setting of two studies was universities [35, 39], one study was conducted at a university care center [23], one was conducted at a fertilization unit of a university hospital [38], one in prisons [36], one in private practice [34], and one was conducted in a semi-residential public service setting [37].

In all seven studies PD treatment was delivered in a group format, of which one study used a semi-structured analytic PD group [37] and one used a protocol for a group PD combined with cognitive-behavioral techniques [36]. All the other studies generally followed the classical PD approach with a standard structure of three PD phases. In three studies, session duration ranged from 1.25 to 3 hours per week, and the number of sessions ranged from 8 to 12 [37–39]. One study delivered six weekly hours for 10 weeks [23]. Two studies were delivered in a concentrated format of a 2- or 3-day intervention [36] and a 2.5-day workshop [34]. Two of the seven mixed methods studies did not provide information concerning the qualifications of the therapists [35, 36]. Only one study indicated that adherence to PD treatment was monitored for treatment fidelity [34] and another study indicated that the PD therapist received clinical supervision during treatment implementation [23].

Qualitative data were collected using post-treatment individual interviews and/or feedback forms in three studies [34–36]. Two studies included direct observation during the sessions in addition to interviews [38, 39], one had focus group interviews both before and after treatment [23], and one presented case study descriptions of clients [37]. Detailed and clear descriptions of qualitative data analysis procedures were absent in all studies, and only one study specified the procedure used for qualitative data analysis as “descriptive analysis” [23]. Quantitative data were collected in all seven studies before and after treatment (pretest-posttest), and one study also collected data at 2-week and 3-month follow-ups [34]. Regarding quantitative data analysis, because of the small sample sizes and/or non-normally distributed variables, four studies computed non-parametric tests: a Mann-Whitney U test for independent samples and/or a Wilcoxon matched-pairs signed-rank test for paired samples [23, 37–39]. Two studies computed *t*-tests [35, 36], of which one also computed a repeated measures analysis of variance [36], and one study computed linear mixed models analysis but without a detailed description of the analysis [34]. Only two studies reported effect sizes. The study with adult inmates reported the highest (medium) effects for improved self-efficacy (*d* = 0.67) and social and friendship skills (*d* = 0.54) [36]. The study on adults with HIV or AIDS reported a large effect size on mental health improvement (*d* = —.82) [23].

In all studies, some context and setting were described but more details could have enhanced the findings' transferability. The recruitment procedure was reported in all studies and information on the sample demographics varied across studies but was satisfactory. Obtaining informed consent from participants was indicated in five studies, with the exception of two [39] of which one only stated that participation was voluntary [35]. The issue of protecting participants’ confidentiality and anonymity was mentioned in one study alone [34] but two other studies referred to the group as a “confidential environment” [23] or to the “confidentiality of the sessions” [35].

Three studies mentioned the potential influence of the therapists or the researchers on the qualitative findings; e.g., through their interactions with clients/ interviewees [23, 34, 36], but only one implemented a procedure: “To reduce potential bias arising from dual roles, protagonists who had been directed by the first author were interviewed by other team members” [34]. In all studies sufficient qualitative data were presented to support the findings, ranging from relatively long quotations [34–36], to short quotations [23, 37, 38], or a combination of both [39]. Procedures to enhance the credibility of the qualitative findings were not reported in any of the studies. Finally, while only two studies were defined as “mixed methods” [38, 39], the collection of both qualitative and quantitative data was appropriate for the purpose of all the studies. In all studies, the integration of qualitative and quantitative data was presented in the Discussion section to a varying degree. No study included an integrated mixed method question or hypothesis.

### Quantitative single group studies

As seen in Table 3, the number of participants in the six single group studies ranged from 12 to 30 (*M* = 21, *SD* = 7.76). Three studies involved children and/or adolescents [29, 40, 41] and two studies involved university students: one on anger management problems in Turkey [42] and one on mental health conditions in Italy [43]. One study consisted of both adults with mental health conditions and students in Israel [44]. The setting of two studies was schools [29, 41], three studies were conducted at universities [42–44], and one study was conducted at an outpatient clinic [40].

In five studies, PD treatment was delivered in a group format, of which one study used a 3-stage protocol for psychoeducation sessions based on PD and some cognitive-behavioral techniques [42], in one group analytical PD was used with a protocol that was not reported [43], and the other four group PD studies followed the standard three PD phase structure. One study was delivered in an individual format, but some sessions were also attended by the adolescent’s or child’s parents [40]. In three studies, session duration ranged from 1.25 to 2 hours per week, and the number of sessions ranged from 16 to 40 [29, 43, 44]. In two studies PD was delivered twice a month [40, 42] and in one study in a 3-day format with unspecified hours [41]. Information concerning the qualification of the therapists was provided in only three of the six studies [29, 43, 44]. One study indicated that the PD director was “one of the researchers” [41] and another study indicated that participants were treated “by the author” [40]. Only one study described a procedure for ensuring implementation fidelity [44] and one study indicated that the PD therapist received clinical supervision during treatment implementation [29].

Data were collected in four studies before and after treatment [40– 43], and one of these studies also collected data at a 22-month follow-up [40]. Two studies had a single case design (also termed “single subject design”) with repeated measures over the course of the treatment, of which one also had three post-treatment measures [44] and one measured in-session changes [29].

Data analysis included a non-parametric Mann-Whitney U test for independent samples and/or a Wilcoxon matched-pairs signed-rank test for paired samples [40, 42]. Analysis of variance and paired *t*-tests were computed in two studies [41, 43]. One study with a single case design computed a hierarchical linear model that took the nested data structure into account and compared aggregated measures at three time points [44]. Another study with a single case design computed a hierarchical linear model and odds ratio, which is an effect size statistic reflecting the odds of clients’ dramatic engagement in predicting their productive in-session behaviors, with effects ranging from small (0.70) to medium (4.30) [29]. No other studies reported effect sizes.

The recruitment procedure was reported in all the studies. Information on sample demographics varied across studies but was generally satisfactory, with the exception of two studies that only reported age and gender [40, 41]. In three of the six studies it was indicated that participants provided informed consent [40, 41, 43].

### Non-randomized group comparison

The number of participants in the two non-randomized group comparison studies ranged from 23 to 40 (*M* = 32, *SD* = 12), as seen in Table 4.

One study involved women coping with the first episode of major depression was conducted at a university polyclinic in Turkey [19]. This study included three groups and compared the effects of group PD with anti-depressant medication to the effects of a group with antidepressant medication alone and to a healthy control group of volunteers with similar ages and education levels. The second study involved adolescents at risk that was conducted at a middle school in Israel [45]. This study included two groups and compared the effects of group PD to a waiting list control group.

Regarding the treatment, the study at the university polyclinic had a loosely structured treatment with a session duration of three hours per week, for 16 sessions [19]. The study at the school did not follow a treatment protocol, and session duration was 1.5 hours per week for 16 to 20 sessions [45]. In both studies, information concerning the qualification of the therapists was provided and both studies indicated that the therapist was given clinical supervision during treatment implementation.

Data were collected in both studies before and after treatment (i.e., pretest-posttest). In one study, due to small sample size and non-normally distributed variables, the data analysis included a non-parametric Kruskal Wallis test for the comparisons of the three groups and a Wilcoxon matched-pairs signed-rank test for paired samples [19].The second study included a mixed‐design repeated measures analysis of variance and process-outcome analyses based on correlations between the change scores for the process and outcome variables [45]. Only the latter study reported effect sizes, with effects ranging from medium (.11) to large (.28) partial eta-squared. In both studies, the recruitment procedure was reported but information on sample demographics was more detailed in one study [45] than the other [19]. In both studies participants provided informed consent.

### Randomized group comparison

As seen in Table 4, the number of participants in the 11 randomized group comparison studies ranged from 16 to 113 (*M* = 53, *SD* = 30). Four studies were on adolescents, of which three were on adolescents with aggression problems in Turkey (reported as distinct studies [46–48]) and one on Latino immigrant adolescents in the US (the same study was reported in two separate articles [21, 22]). The remaining seven studies were on adult smokers in Turkey [49], men with opiate dependence in Iran [50], adults with burnout who work in public services in Finland [18], oncology nurses coping with burnout in Turkey [51], adults with Parkinson’s disease in Brazil [52], and adults with panic disorders in Bulgaria [30]. One study was on university students with low subjective well-being and high hopelessness in Turkey [53].

The settings of three studies were schools [46–48], three studies were conducted at health service centers [18, 30, 50], two studies at hospital clinics [51, 52], and two at universities [49, 53]. One study was conducted in community settings [21, 22].

In all studies PD treatment was delivered in a group format, of which five followed the standard three PD phase structure [46–48, 50, 53]. Two treatments were semi-structured [49, 51], one treatment had a structured protocol [21, 22], whereas in three studies the content of the PD treatment was unclear [18, 30, 52].

In most studies (n = 8 of 11, 72%), session duration ranged from 1.5 to 3 hours per week, and the number of sessions ranged from eight to 25 [21, 22, 30, 46–49, 51, 53]. One study delivered a 2-hour group PD for 12 sessions over six weeks [50], another delivered 6-hour group PD sessions over 17 days [18], and in one study a 1.5-hour group PD was delivered twice a month for 12 sessions [52].

Therapists’ qualifications were reported in eight studies, of which only six reported that the PD groups were led by psychodramatists [22, 30, 46, 49, 51, 53] and two studies reported that the PD groups were led by a psychologist and a physiotherapist [18], and a psychologist [52]. The remaining three studies did not report any information about the therapists’ qualifications [47, 48, 50]. Only two studies indicated the PD therapist received clinical supervision during treatment implementation [22, 46].

Data collection in most (9 of 11, 81%) of the randomized group comparison studies included before and after treatment measures and a follow-up measure that ranged from 10 weeks to 1 year after the treatment, with the exception of two studies that only included pretest-post measures [50, 52]. Data analysis in most studies (7 of 11, 64%) used parametric tests such as analysis of variance and/or *t*-tests, [18, 30, 46, 47, 50–52], as well as regression analysis [21, 22]. Three studies used non-parametric tests, including a Mann-Whitney U test for independent samples, a Wilcoxon matched-pairs signed-rank test for paired samples, and/or a Kruskal-Wallis test for independent samples, a chi-square test or a Fisher exact test for categorical data [48, 49, 53]. Three studies reported moderate to large effect sizes [18, 21, 22, 50], and one study reported an odds ratio indicating a small effect size [49].

The recruitment procedure was reported in all studies and information on sample demographics varied across studies but was generally satisfactory, with the exception of five studies that only reported age (or grade), gender, and/or scores on a screening instrument [18, 46–48, 53]. In two studies it was indicated that participants provided informed consent [51, 52] and two studies indicated that participants volunteered [46, 47, 53].

### Risk of bias assessment

**Fig 4:**
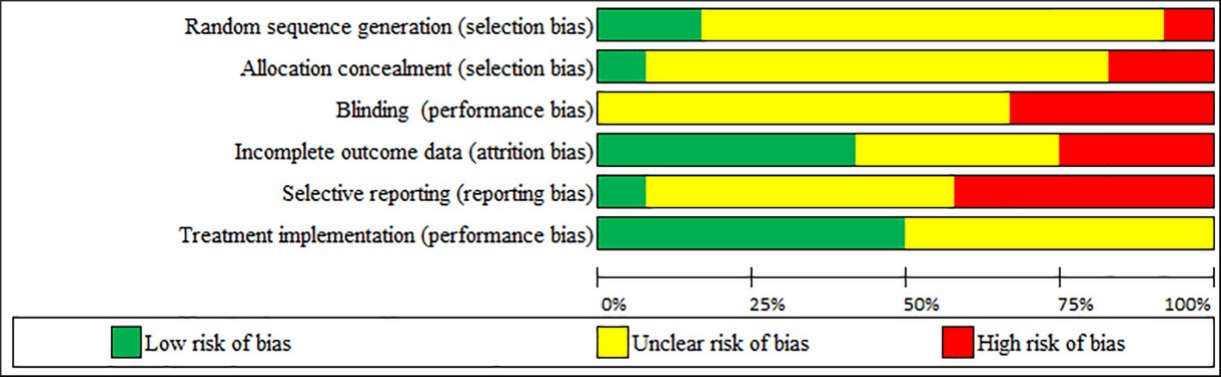
Proportion of studies for each of the six potential sources of bias.

This section reports the risk of bias analysis results for 12 randomized group comparison studies (11 in Table 4, and the Dogan et al. 2010 study in Table 2). A summary of the risk of bias analysis is presented in Table 5 and the proportion of studies for each of the six potential sources of bias is presented in Fig 4.

**Table 5:** Risk of bias assessment results per study.

First author (year)	Random sequence generation (selection bias)	Allocation concealment (selection bias)	Blinding (performance bias)	Incomplete outcome data (attrition bias)	Selective reporting (reporting bias)	Treatment implementation (performance bias)
Aytemur (2012)	**Low**	**Low**	**Unclear**	**Unclear**	**Unclear**	**Low**
Dehnavi (2016)	**Unclear**	**Unclear**	**High**	**Low**	**Unclear**	**Unclear**
Dogan (2010)	**Unclear**	**Unclear**	**Unclear**	**High**	**Low**	**Low**
Kähönen (2012)	**High**	**High**	**High**	**High**	**High**	**Unclear**
Karataş (2011)	**Unclear**	**Unclear**	**Unclear**	**Low**	**High**	**Unclear**
Karataş (2014)	**Unclear**	**Unclear**	**Unclear**	**Low**	**High**	**Low**
Karataş (2009a)	**Unclear**	**Unclear**	**Unclear**	**Unclear**	**Unclear**	**Unclear**
Karataş (2009b)	**Unclear**	**Unclear**	**Unclear**	**Unclear**	**Unclear**	**Low**
Özbaş (2016)	**Low**	**Unclear**	**High**	**Unclear**	**High**	**Low**
Smokowski (2009a,b)*	**Unclear**	**Unclear**	**Unclear**	**High**	**Unclear**	**Low**
Sproesser (2010)	**Unclear**	**Unclear**	**High**	**Low**	**High**	**Unclear**
Tarashoeva, (2017)	**Unclear**	**High**	**Unclear**	**Low**	**Unclear**	**Unclear**

Risk of bias assessment was performed only for the 11 studies with randomized group comparison, corresponding to Table 4 and Dogan (2010) in Table 2.

*The results of the same study were reported in two separate articles.

As can be seen, none of the studies was identified as having a low risk of bias across all six sources of bias. Evidence for potential *selection biases* (both “random sequence generation” and “concealment of allocation to conditions”) were unclear in the majority of studies (75%, 9 of 12). One study was at low risk of these two selection biases because it had a restricted block randomization method and used sealed envelopes “prepared before enrollment by the head nurse” [49], suggesting that participants and investigators enrolling participants were blind to the allocation to conditions. A high risk of these two sources of selection biases was evident in a study where clients were randomized “into the two different intervention groups but not into the control group” [18]. Thus, randomization was incomplete and clients or investigators could have possibly foreseen the allocation to conditions. Another study was at high risk for inadequate allocation concealment because it was explicitly defined as “an open” clinical study in which the allocation to conditions was not concealed [30].

The blinding of participants and personnel (i.e., clients and therapists) is intended to prevent the direct and indirect influence of their expectations or motivations on the study outcomes. However, this type of *performance bias* is impossible to avoid in psychotherapy: therapists know what treatment they are delivering, and clients not only are aware of receiving that treatment but also have expectations concerning the treatment [27]. For this reason, psychotherapy reviews have considered there is a low risk of bias on the basis of two adjusted criteria: (a) the use of an active control group with a credible treatment involving comparable treatment expectancies, and (b) the assessment of client-perceived “treatment expectancies or credibility prior to or early (i.e., after the first session) in treatment and in which the treatment group had equal (zero difference) or lower expectancies or credibility than the control group received” (see Appendix C, p. 4, in [28]). In the present review, none of the studies was identified as having a low risk of performance bias according to these criteria. Evidence for potential blinding performance bias was unclear in the majority of studies (67%, 8 of 12) that had an active control group but did not measure client-perceived treatment expectancies or credibility. Four studies (33%) were at high risk of performance bias because they had an inactive control group and no measures of treatment expectancies or credibility [18, 50–52].

*Attrition bias* refers to the mishandling of incomplete outcome data. There was a low risk of attrition bias in five studies (42%) with no reported dropouts, appropriate imputation of missing data, and/ or unlikeliness that the missing data were related to the outcome [30, 48, 50, 52, 53]. The risk of attrition bias was unclear in four studies (33%) due to insufficient reporting of attrition [46, 47, 49, 51]. There was a high risk of attrition bias in three studies that had a dropout rate exceeding 20% and no imputation of missing data [18, 21, 22, 39].

Selective outcome *reporting bias* refers to cases where outcomes that were collected according to the methods section (or to a pre-study published protocol or a clinical trial’s registry) are not reported. There was a low risk of reporting bias in only one study [39], and an unclear risk in five studies [21, 22, 30, 46, 47, 49, 50]. There was a high risk of reporting bias in five studies where not all of the pre-specified primary outcomes or not all of the results related to the outcome were reported [18, 48, 51–53].

Treatment implementation is a type of performance bias that refers to deviations from intended treatment that are likely to have affected the outcome and to the qualifications of the therapist delivering the treatment [27]. There was a low risk of *treatment implementation bias* in six studies because both the psychodramatists’ qualifications and the treatment content were reported [22, 39, 46, 49, 51, 53]. The remaining six studies were at unclear risk of treatment implementation bias because the qualifications of the therapist were not reported [47, 48, 50], the therapist was not identified as a psychodramatist [18, 52], and/or a psychodramatist delivered the therapy but no content was described [30]. None of the studies was identified as having a high risk of treatment implementation bias.

## Discussion

This is the first integrative review to systematically examine methodological issues in 31 intervention studies on PD psychotherapy. Overall, the studies examined the effect of PD on more than 20 different outcomes and most studies had adult clients. The next largest group was adolescents, and only two studies involved children.

In reviewing PD intervention studies, it is important to consider its core theoretical concepts of spontaneity and creativity. Spontaneity refers to the pro-creative, catalyzing state of readiness by which creativity emerges, whereas creativity refers to the creative act itself; namely the creative, cognitive, emotional, behavioral response or a tangible creative product [54]. Moreno considered creativity essential to adapting to life changes and to coping with unexpected challenges [55]. This notion corresponds to contemporary views of creativity as an important factor for adaptation [56, 57]. Relatedly, Moreno associated mental health with the ability to enact a wide repertoire of roles that enable the individual to act flexibility and adequately, in the right way at the right time [58]. Thus, it is striking that only one study included in this review measured spontaneity [30] and that none measured creativity or other PD constructs. To substantiate PD as a distinct treatment modality, future intervention studies would do well to measure such modality-specific constructs that might account for treatment effects based on psycho-dramatic theoretical reasoning.

In light of these findings, in the next sections we recommend specific guidelines to improve the methodology, transparency, and specificity in reporting PD, or other intervention research. The proposed guidelines build on several resources in psychology [59, 60], psychotherapy [27], and music therapy [61].

### Qualitative studies

The four qualitative studies presented sufficient excerpts from the data which showed that overall, PD can contribute to clients’ self-worth and promote self-awareness, self-expression, and better perceived coping with difficulties. The descriptions of participants’ characteristics, the contexts, and settings could have been more detailed to allow for comparison with other contexts and settings and to enhance the transferability of the findings. According to the American Psychological Association’s new Journal Article Reporting Standards (“APA JARS”) for Qualitative Research, “transferability of findings in qualitative research to other contexts is based on developing deep and contextualized understandings that can be applied by readers” [60]. The rationale for choosing a specific data analysis method varied across studies and, generally, descriptions could have been clearer and more detailed. In addition, the researchers did not provide a reflexive self-description of their views and backgrounds (e.g., demographic/cultural characteristics, values, experience with the phenomena, training, etc.), or whether and how reflexivity was used to mitigate their potential influences on the participants and/or the interpretation of the findings. It is worth noting that one study applied a procedure to enhance the credibility of the findings by confirming the emergent themes with a psychodrama expert [32]. Future studies could enhance the credibility of the findings by implementing procedures such as data triangulation, disinterested peer debriefing, external auditing, or member checking (also called “informant feedback” or “respondent validation”).

### Mixed methods studies

Mixing qualitative and quantitative methods strengthens the internal and external validity of the findings, in that the advantages of each method complement one another, and their shortcomings are considerably offset [62]. In the seven mixed methods studies, data integration was presented in the Discussion section, which was useful for interpreting the findings based on the two datasets as well as for giving a (qualitative) voice to participants and ensuring that the quantitative results reflected their experiences. An “integrated” mixed method question or hypothesis which directly addresses the mixing of the quantitative and qualitative strands of the study were not put forward in any of the studies. An example of an integrated question that conveys the *methods* of the study is “Do the qualitative data help explain the results from the initial quantitative phase of the study?” and an example that conveys the *content* of the study is “Do clients identify helpful aspects in therapy that shed light on why or how therapy was effective?” In addition, most studies did not contain a statement about the rationale for choosing a mixed methods design such as a more comprehensive and nuanced understanding of the research problems. Researchers should also be encouraged to state the type of mixed methods design used, and specify the timing of the data collection (concurrent or sequential) as well as the emphasis (equal or unequal) on each data type. Examples of mixed method designs include Convergent Parallel design, Exploratory Sequential design, and Explanatory Sequential design [63]. One mixed methods approach that has been adopted by some PD researchers in Europe [64] is the Hermeneutic Single-Case Efficacy Design which integrates quantitative outcome and weekly change data with clients’ qualitative accounts of change over therapy, while examining alternative non-therapy explanations for changes in therapy [65].

### Quantitative studies

#### Single group

Quantitative studies with a single group pretest-posttest design are sometimes the only viable option in applied research that occurs in natural field settings. This design is quasi-experimental and some consider it uninterpretable given the multiple threats to internal validity associated with the lack of a control group [66]. Although this design allows the researcher to determine whether a change occurred between pretest and posttest, this change may be attributed to factors other than the treatment, such as history (i.e., an event that can impact the outcome) or maturation (i.e., clients’ natural growth or development). Thus, results of studies with a single group pretest-posttest design have less certainty.

When a control group is unavailable, studies with a single case design can provide more certainty on whether the intervention is responsible for change because this design involves multiple measures over the course of the treatment, with each client serving as his or her own control for purposes of comparison [67]. Single case design is underused in PD research although it is widely employed in applied field studies when a control group is unavailable. Experts have stated that single case designs can “provide a rigorous experimental evaluation of intervention effects… [and] a strong basis for establishing causal inference” [68]. Established standards for single case designs require a minimum of five data points in a phase (and at least three phases) to meet evidence standards without reservations [68]. For example, five systematic measures before (i.e., at baseline), during, and after the treatment may demonstrate an effect when the data pattern in the treatment phase differs from the data pattern observed in the baseline phase.

#### Nonequivalent groups

The four studies (of which two were mixed methods) with a non-randomized group comparison design represent another type of quasi-experimental study that is considered more interpretable than the single group pretest-posttest design [66]. Studies with a non-randomized group comparison design typically include nonequivalent groups that can differ on various characteristics due to the lack of randomization. In particular, two of the studies included in this review had a control group of participants who were matched on several characteristics to control for pre-treatment differences that could introduce selection bias [19, 37]. In studies with a non-randomized group comparison design, matching or examination of pretest equivalence can increase confidence in attributing any observed posttest differences between groups to the treatment rather than to some pretest differences. In addition, researchers are encouraged to employ statistical procedures that account for pre-treatment differences such as using the pretest score as a covariate in analyzing the posttest scores [69].

#### Randomization

Of the studies included in this review, 48% (*n* = 12) had a randomized group comparison design. To reduce selection bias, researchers may choose to allocate participants randomly to conditions with a computerized random number generator or a table of random numbers, etc. rather than a systematic but non-random approach (e.g., birth or admission dates, record number). The allocation to conditions should be concealed so that study personnel who enroll participants remain blind to the condition assignment, for example by using sequentially numbered, opaque, sealed envelopes. This can prevent study personnel from excluding or changing allocations of particular clients that can undermine randomization and “might cause baseline differences in characteristics relevant to outcomes” [27].

#### Control groups

Five group comparison studies included an active control group (“active comparator”) that was used to examine the specific effect of PD treatment compared to other acknowledged psychotherapies or a placebo activity. Most group studies, however, compared PD to standard care or passive control with no treatment. Therefore, the specificity of the PD effects remained equivocal in most studies. As the current evidence suggests that PD has an effect, an important next step would be to examine whether this effect is due to specific “active ingredients” in PD or more general common factors that are shared across therapeutic modalities [70]. The use of a placebo or an active control group with a credible treatment can also minimize performance bias related to the impossibility of blinding therapists and clients in psychotherapy research by controlling for treatment expectancies that may influence the outcome [27].

#### Mediation and moderation

Mediation and moderation are being increasingly explored in psychotherapy research that aims to go beyond basic questions about effectiveness [71, 72], but is still rare in PD research. Hypothesizing mediators (i.e., a variable that accounts for the indirect association between an intervention and outcome) can shed light on “why” or “how” therapy leads to change, which ultimately can facilitate the optimization of change [73, 74]. To date, there are no evidence-based explanations of precisely why psychodrama works and how it leads to changes. Future studies could pinpoint modality-specific mediators that might account for treatment effects based on theoretical reasoning and a scientific rationale for why the specified PD component (e.g., role-reversal) might influence the outcome of interest (e.g., empathy). Similarly, hypothesizing moderators (i.e., a construct that influences the direction or magnitude of the relationship between the intervention and outcome) can lead to a better understanding of “when” or “for whom” therapy leads to change [75]. Moderators are often client or therapist characteristics (e.g., sex, ethnicity, temperament) or the treatment delivery format (e.g., individual vs. group treatment) [73, 74]. Mediators and moderators should be clearly articulated in the theoretical model upon which the treatment is based to establish “treatment differentiation” that clarifies how the PD treatment differs from the comparison or control group.

#### The PD treatment

Clear descriptions of treatment duration, frequency and settings varied across studies. It is particularly important to provide justifications when examining PD treatments that are unique in delivery format, such as when PD is delivered in an intensive format or with a considerable gap between sessions (i.e., weeks or months). Of the studies included here, 54% (*n* = 16) employed the three standard phases of a PD session (warm-up, action, and sharing). The remaining studies had semi-structured treatments (*n* = 6, 20%), a structured protocol (*n* = 4, 13%), or an unclear /unspecified structure (*n* = 4, 13%). Replication and transparency can be promoted by clearly reporting the objectives and the content of the sessions, appending the treatment protocol where applicable, and by specifying the essential active components and how they are incorporated into the PD treatment. In any case, it is important to define PD-specific terminology for international cross-disciplinary readership.

#### Implementation fidelity

Treatment implementation fidelity was monitored in three studies that used video recordings [22, 34, 44]. Implementation fidelity is a concern for rigorous research and includes two main components. One is *treatment integrity* that refers to (a) the extent to which a therapist delivers the intervention with adequate *adherence* to the manual and/or intended treatment modality and its theory-specified techniques or methods, and (b) the *competence*, or skillfulness, with which these techniques or methods are implemented. The second component of implementation fidelity is *treatment differentiation* that refers to the extent to which a treatment differs from a comparison or control condition [76]. Because clinical research is designed to compare the treatment modality rather than the therapist’s ability, variations in therapists’ competence and adherence must be minimized and treatment differentiation must be maximized. Thus, observational measures of video/audiotaped sessions or self-report implementation checklists can be used for the ongoing review of implementation fidelity [77].

Relatedly, because PD is not a manualized treatment, it is important to establish an operational definition of PD treatment during the research design phase. Then, during treatment, competent adherence to this definition should be monitored. For example, (a) enactment of at least one scene that approximated a real-life situation or was an externalization of the client’s inner experience; (b) application of at least one of the four fundamental PD techniques (soliloquy, doubling, mirroring, role reversal, and concretization), and (c) the fact that group members played roles in the client’s enactment [34, 78]. In applied research, as long as there is competent adherence to the essential active components of a treatment, the treatment can be somewhat flexible and adaptable according to clients’ needs or external conditions. Overall, the need for monitoring implementation fidelity calls for the development of measures that focus on PD therapists’ competence and adherence, similar to other therapies [79].

#### Therapist training

Another important component of implementation fidelity is therapist training, selection, and clinical supervision to monitor delivery quality. “Psychodrama” is a profession-specific term that should not be used to describe treatment delivered by healthcare professionals who do not have PD credentials. Because this issue influences the quality of delivery directly, the PD practitioner should be adequately trained to deliver the treatment and subjected to assessment and ongoing evaluation and supervision. Information on specific theoretical orientation and the therapists’ pre-implementation training should also be provided. Seven (23%) of the studies here indicated that the therapists received clinical supervision during treatment implementation. Overall, more information on the qualification level of the therapist who applied the PD is needed, since only 58% of the studies (*n* = 18) reported that the treatment was delivered by a trained psychodramatist.

#### Handling missing data

Almost half of the studies had a low risk of attrition bias. Non-respondents and clients’ early dropout from treatment leads to incomplete data. To minimize attrition bias due to incomplete data, researchers can choose from several methods for handling missing data [80]. Researchers are encouraged to use both *intent-to-treat analysis* (ITT), in which all participants initially enrolled in the treatment are included in the group to which they were initially assigned by statistically imputing (replacing) missing data with substituted values, as well as *completer analysis* in which only clients who completed the treatment are included in the analysis and no data imputation is performed. It is important to note any difference in results for the effect of treatment “as assigned” (ITT) versus treatment “as treated” [81, 82].

#### Effect size and statistical power

Statistical significance measures how likely it is that differences in outcome between treatment and control groups are real and not due to chance. The most commonly used measures of statistical significance are *p* < .05 and 95% confidence intervals (CI) that “estimate the range within which the real results would fall if the trial is conducted many times” [83]. But while measures of statistical significance can inform the reader as to whether a difference or an effect exists, they cannot reveal the size of the effect. In other words, the effect size indicates not only whether a treatment affects clients, but how much it affects them [84]. Unlike a *p* value, effect size is independent of sample size. Common effect size indices are Cohen’s *d* for the comparison of group means (e.g., *t*-test) and the partial eta-squared for the analysis of variance [85]. Most of the quantitative studies (67%) reported the statistical significance of the findings but did not report effect size, which is important in determining the magnitude of the difference between groups.

Another procedure that is underused in PD research is the calculation of statistical power, which is defined as the probability of avoiding a Type II error; i.e., rejecting the null hypothesis of no effect when in fact there is an effect. A-priori calculation of statistical power can help researchers determine the optimal sample size for testing their hypotheses during the design phase of their study. A reasonable minimum level of power to aim for is .8 [86].

A related concept to consider is the *clinical significance* of the findings (also termed clinical or practical importance) which refers to “the extent to which therapy moves someone outside the range of the dysfunctional population or within the range of the functional population” [87]. Thus, clinical significance refers to the practical importance of treatment in terms of actually reflecting a clinically meaningful change. In some cases, a large sample size may yield a statistically significant difference between groups that has little clinical significance (i.e., clinically meaningful change) and in other cases a non-statistically significant result may fail to detect an important and meaningful difference between groups; e.g., due to an underpowered study with a small sample. Researchers are encouraged to employ statistical methods of analysis that estimate clinical significance in psychotherapy research [88, 89]. Finally, it is imperative to report all of the study’s pre-specified (primary and secondary) outcomes, including non-significant results, to avoid selective reporting bias. A rationale should be provided if researchers only report the total score of a scale that includes distinct subscales scores mentioned in the Introduction or Method sections.

## Conclusions

This integrative systematic review sought to produce a comprehensive summary of the state of PD intervention research in the last decade as well as to highlight and critically evaluate methodological issues that can inform future quantitative, qualitative, and mixed methods studies. The limitations of this review include the exclusion of non-English studies and articles published in non-indexed journals. Overall, the review suggests that PD intervention research in the last decade has followed an upward trajectory, and reports promising results across methodologies. This review constitutes a call to improve methodological and reporting quality. Advances are also needed in theorizing and examining PD modality-specific mechanisms of action or change–which distinguish PD from traditional talk psychotherapy–and their impact on desirable outcomes. The current findings should not be viewed as discouraging because although PD is a long-standing experiential psychotherapy that originated in the beginning of the 20th century, it is an emerging sector in the psychotherapy research arena. As PD research becomes more focused and methodologically rigorous, meta-analytic reviews will provide empirical indices of documented effects. Meanwhile, we encourage authors, journal editors, and reviewers to consider our recommendations for PD intervention research. Further improvement in the methodology, transparency, and specificity of reporting PD intervention research is important not only for scientific purposes but also for the professional status of PD and quality of care.

## Supporting information

S1 Table PRISMA checklist. (PDF)
